# Environmental life cycle of fentanyl: From the cradle to an unknown grave

**DOI:** 10.1002/jeq2.70016

**Published:** 2025-03-24

**Authors:** Deseree J. Reid, Kaizad F. Patel, Angela M. Melville, Vanessa L. Bailey, Kristin M. Omberg, Loreen R. Lamoureux

**Affiliations:** ^1^ Chemical and Biological Signature Sciences, Pacific Northwest National Laboratory Richland Washington USA; ^2^ Biological Systems Science, Pacific Northwest National Laboratory Richland Washington USA

## Abstract

The lack of available information on the presence and persistence of fentanyl in the environment is a significant gap in the technical literature. Although the origins of the opioid in the environment are well‐known because they follow the same pathways of other drug‐related environmental contaminants, the downstream effects of fentanyl in the water supply and its retention in soil are less understood. The characterization of fentanyl and its potential degradation products in complex environmental samples such as soil is severely understudied. Very few articles are available that work to identify fentanyl and its degradation products in complex samples or name the possible hazards that may result from environmental exposure and degradation. Therefore, the objectives were to identify available articles focused on environmental fentanyl and its pathways and highlight quantifiable research or results that included specific degradation products or downstream effects. Research articles focused on fentanyl between 2000 and 2024 were identified and reviewed and then filtered using Boolean search terms for environmental parameters. Various studies have determined that trace levels of fentanyl can be found in a variety of environments, and additional data suggest preferential partitioning into soils from water and long‐term persistence. Despite this knowledge, very little data exists on the long‐term downstream effects of fentanyl or its analogs. As the chronic effects from low‐level fentanyl exposure are currently unknown, this lack of insight brings to the forefront the need for further research to improve our understanding of fentanyl persistence, degradation, and toxicity within the environment.

## Core Ideas

- Fentanyl is introduced into the environment in a variety of ways.
- Fentanyl is increasingly detected in environmental samples.
- Degradation products of environmental fentanyl are largely understudied.
- Potential chronic effects of environmental fentanyl exposure are unknown.

**Abbreviations**
EC: emerging contaminant
EPA: Environmental Protection Agency
WBE: wastewater‐based epidemiology

## 1 INTRODUCTION

**FIGURE 1:**
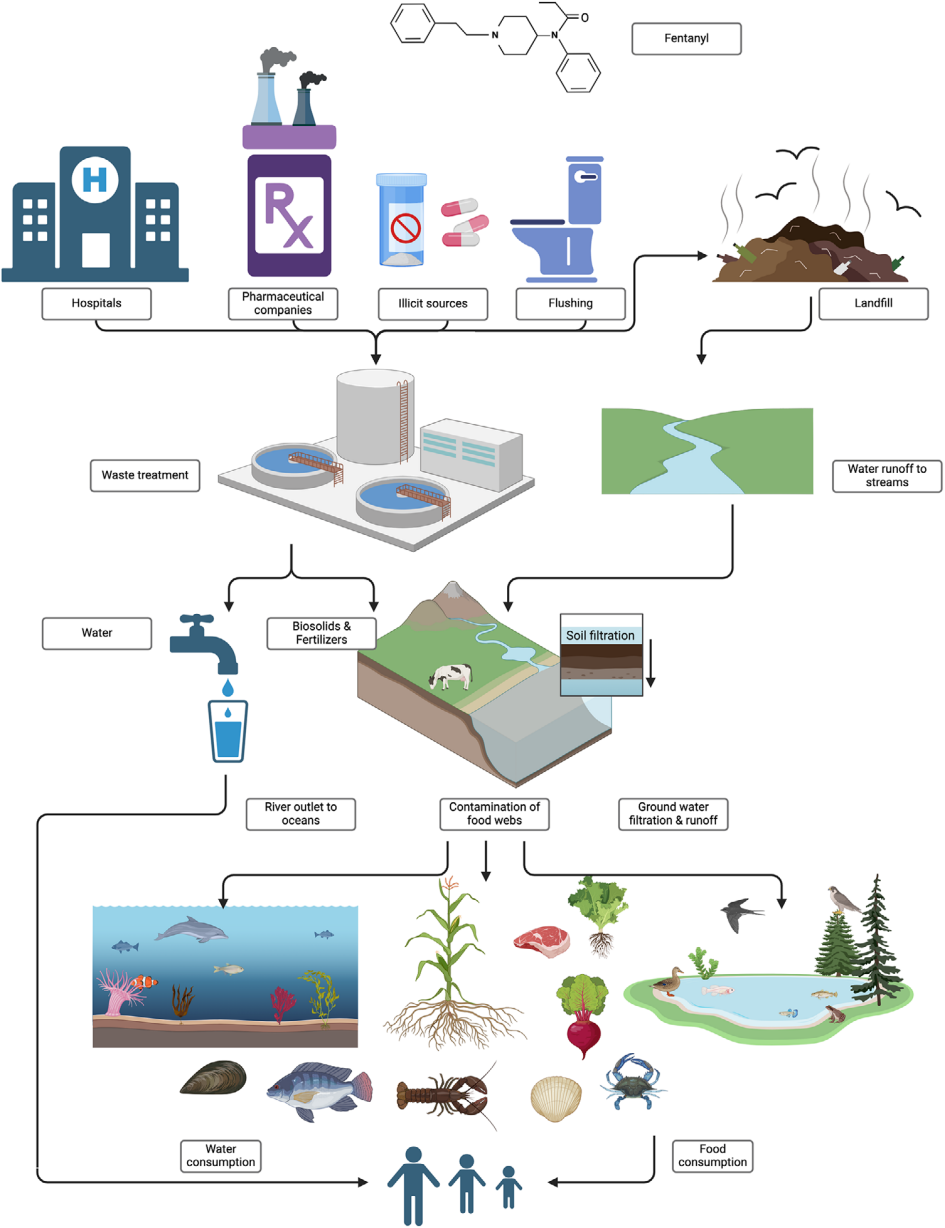
Environmental fentanyl originates predominantly from households, pharmaceutical companies, illicit producers, and healthcare facility waste products. Solid and liquid remnants used in production end up in landfills or as fertilizers. Ultimately, water runoff and waste treatment plants distribute the contaminant into the water table, where it contaminates agriculture and livestock, making its way into the food chain. (The figure was created in BioRender; https://BioRender.com/).

Fentanyl, like many pharmaceuticals, personal care products, and illicit drugs, has been found in sewage and wastewater treatment plants, and in ground, surface, and drinking waters around the world (Castiglioni et al., 2008; Fick et al., 2009; Yu et al., 2020). Despite this, little is known about the long‐term effects of environmental contamination and the biodegradation, or lack thereof, of fentanyl. When pharmaceuticals, drugs, and other compounds are distributed through aquatic environments, they may accumulate in the tissues of wildlife such as insects, birds, fish, and their predators (Figure 1) (Ebele et al., 2017; Muir et al., 2017). Pharmaceutical environmental contamination has been shown to alter the endocrine systems of aquatic organisms (Benotti et al., 2008; Colborn et al., 1993) and contribute to antimicrobial resistance (Baquero et al., 2008; Williams‐Nguyen et al., 2016), which suggests the potential for eventual impacts on human health. While antibiotics, endocrine disruptors, and microplastics are the most studied environmental contaminants, there is growing concern about unregulated chemicals, such as pharmaceuticals, drugs, personal care products, detergents, pesticides, or agricultural products, also referred to as emerging contaminants (ECs). The United States Environmental Protection Agency (EPA) defines an EC as a chemical or material characterized by a perceived, potential, or real threat to human health or the environment, or by a lack of published health standards (United States Environmental Protection Agency, 2023).

Fentanyl is a well‐known synthetic analgesic and an EC of growing concern due to its potency and number of equally or more toxic analogs. As little as 2.0 mg of fentanyl can be lethal to a human. Fentanyl has been detected in sewage effluent in the Netherlands (van der Aa et al., 2013), river sediments in Croatia (Babic et al., 2018), wastewater in the United States (Croft et al., 2020; Gushgari et al., 2018, 2019) and Australia (Tscharke et al., 2016), wastewater treatment plant effluent and drinking water plants in Canada (Rodayan et al., 2016), and in hospital effluents in Taiwan (Lin et al., 2010), among others. Yet, despite the increasing use of fentanyl and its high potential for bioaccumulation and persistence (Mankes & Silver, 2013), information is sparse regarding its environmental fate and long‐term exposure risks. This review aims to summarize current knowledge regarding the presence, persistence, and potential hazards of fentanyl and its analogs, precursors, and degradation products in the environment.

### 1.1 History and properties of fentanyl

First introduced in the 1960s, fentanyl is 50–100 times more potent than morphine and was originally used in combination with other intravenous hypnotics, sedatives, and amnestics to achieve anesthesia in patients (Schueler, 2017; Stanley et al., 2014, 2008). It was eventually approved for pain management, and the development of other fentanyl‐like compounds and alternative delivery systems soon emerged. By the 1990s, fentanyl was commonly prescribed, and the number of overdose deaths from narcotic analgesics began to rise. According to the United States Centers for Disease Control and Prevention, this initial rise in prescription opioid mortality marked a period now widely regarded as the “first wave” of the opioid epidemic in the United States (CDC, 2024; Ciccarone, 2019). The second wave began around 2010 with increased heroin‐related deaths, which was then shortly followed by the third wave, when dramatic increases in opioid overdoses began to be reported. A significant proportion of these incidents involved illicitly manufactured fentanyl (Ciccarone, 2017; Jannetto et al., 2019). Similar trends were also observed in the European Union (Jannetto et al., 2019). The ease of production and low cost of fentanyl have resulted in it becoming a common adulterant in many illicit substances (Singh et al., 2020).

Fentanyl (*N*‐phenyl‐*N*‐[1‐(2‐phenylethyl)piperidin‐4‐yl]propanamide) is a heterocyclic tertiary aliphatic amine with two different phenyl rings and an amide of aniline, making it chemically similar to morphine and heroin. The free base form is nonpolar and hydrophobic and readily crosses cell membranes. Its pKa varies with temperature (Thurlkill et al., 2005), and at biological pH and temperatures, it is protonated and charged and thus slightly more water soluble than the free base form. The strong analgesic activity of fentanyl is due to its high affinity for the mu opioid receptor (Herman et al., 2023; Lipinski et al., 2019; Vo et al., 2021), and the rapid onset of analgesia after administration results from high lipid solubility and blood‐brain barrier penetration.

As a drug, fentanyl is commonly administered orally and intravenously, but other delivery methods such as sublingual, transmucosal, and transdermal are also utilized, creating a market for lozenges, nasal sprays, and extended‐release patches (Lane, 2013; Grape et al., 2010). While fentanyl can be absorbed through the skin, the very low dermal absorption rate in its dry form means it is unlikely to pose adverse risks via this route (Attaway et al., 2021). Signs of fentanyl overdose are rapid and similar to those of other opioids, with severe onset respiratory depression, apnea, miosis, stupor, and death (Fulton et al., 2022; Jannetto et al., 2019; Somerville et al., 2017; Stanley, 2014).

### 1.2 Research publications on environmental fentanyl

**FIGURE 2:**
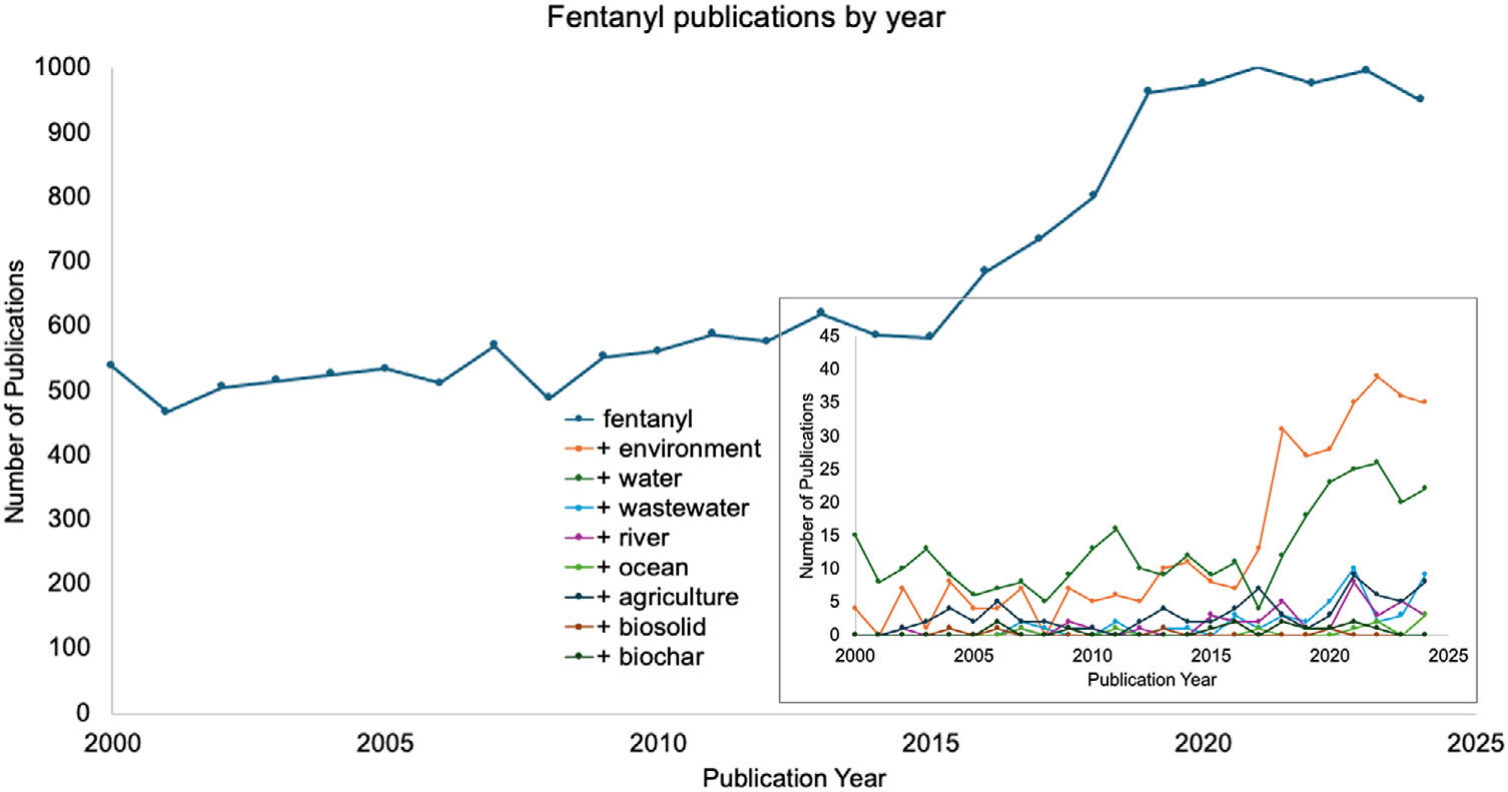
Fentanyl‐related publications graphed by year and by topic. Primary large graph shows all fentanyl‐related publications for a given year between 2000 and 2024. Inset graph (lower right) depicts a reduced *y*‐axis range to facilitate improved visualization of a breakdown of the specific environmentally related fentanyl publications over time.

Currently available data suggest that fentanyl is environmentally persistent and moderately stable (Xega et al., 2020). While incidental exposure to fentanyl at the levels currently detected in environmental samples is unlikely to be acutely harmful, it is not clear if chronic environmental exposure will result in ill effects to aquatic ecosystems, wildlife, and human health (Yadav et al., 2017). To review the available literature on fentanyl in the environment, we utilized Boolean searches in Web of Science to evaluate the number of publications between 2000 and 2024. Results of these searches can be visualized in Figure 2. The initial search parameters were solely on fentanyl, and subsequent searches paired the word fentanyl with the words environment, water, wastewater, river, ocean, agriculture, biosolid, and biochar to eliminate irrelevant results. Between 2000 and 2015, the number of yearly publications on fentanyl remained relatively stable between 500 and 600 per year but then saw an average year over year increase until 2021 where it plateaued and remained relatively stable. When looking at sub‐topics on fentanyl research, the top categories of fentanyl publications involved the environment and water (Figure 2, inset), with publications on fentanyl+water being more prevalent than fentanyl+environment, until 2017 when environmental research publications began increasing. However, the total publication rate of environmentally related articles that evaluate fentanyl levels remains below approximately 100 articles per year, indicating a stark gap in the technical knowledge for such an abundant EC.

**Core Ideas**

- Fentanyl is introduced into the environment in a variety of ways.
- Fentanyl is increasingly detected in environmental samples.
- Degradation products of environmental fentanyl are largely understudied.
- Potential chronic effects of environmental fentanyl exposure are unknown.

**FIGURE 3:**
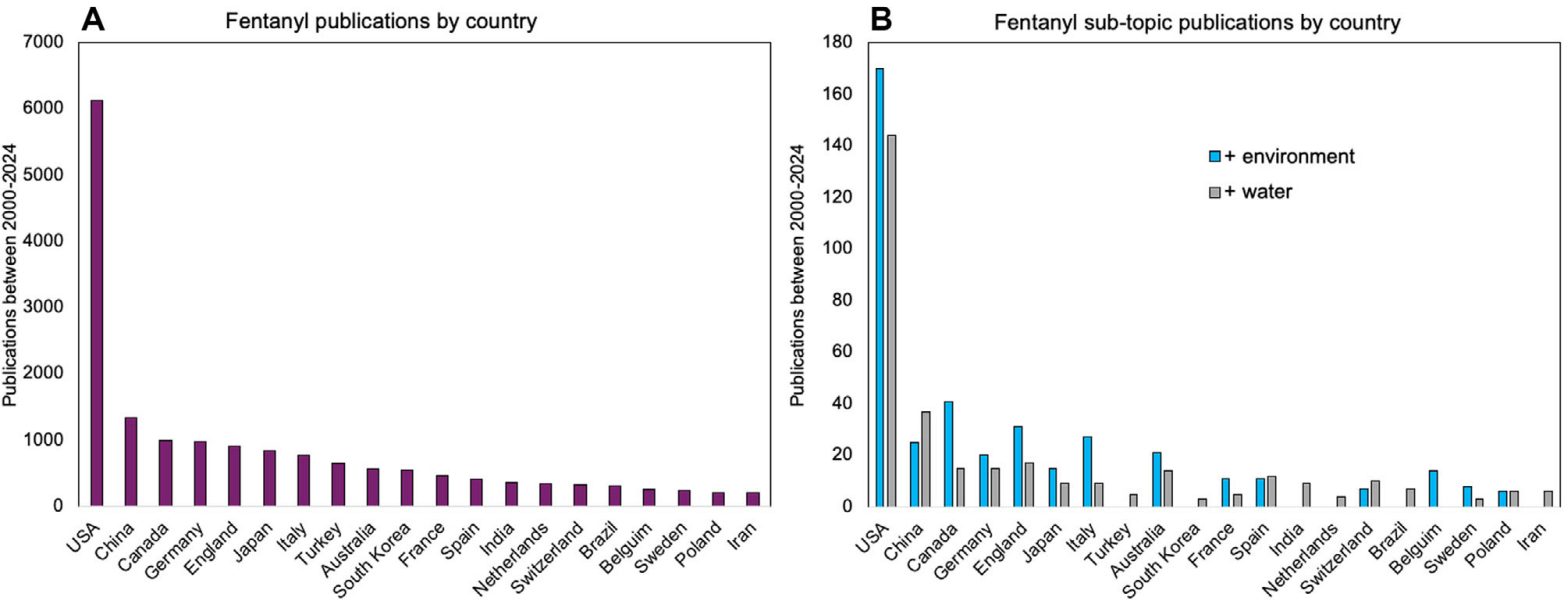
Fentanyl publications by country between 2000 and 2024. (A) Countries with >200 fentanyl publications between 2000 and 2024. (B) Breakdown of fentanyl publications relating to the environment or water in the same countries seen in plot A.

The breakdown of countries that are publishing fentanyl research related to the environment and water systems is further illustrated when looking at the graphs in Figure 3. Geographically speaking, the U.S. published the most fentanyl‐related research overall with 6118 publications (Figure 3A), but the number of overall US fentanyl‐related publications pertaining to the environment or water systems over 24 years was still quite low at 170 and 144 articles, respectively (Figure 3B). China was responsible for the most literature after the United States (1341 articles), but only 25 and 37 respective articles were related to the environment and water systems. In comparison, China published 4.6% of their fentanyl research on environmental fentanyl, while the US segment was 5.1%, indicating a relatively equivalent interest in environmental implications. All other countries cumulatively produced <500 articles over 24 years on environmental fentanyl, or fentanyl in water systems. Based on the data that indicate increasing usage of fentanyl for legitimate and illegitimate purposes, it is critical to understand the full pathways of fentanyl from synthesis to disposal and degradation.

The low number of publications related to environmental fentanyl and its degradation products is especially problematic for predicting long‐term environmental outcomes. This significant knowledge gap is likely partially due to difficulty in accurate environmental sampling as procedures vary widely by contaminant and the medium being sampled (Washington State Department of Health [WSD Health], 2005; Organization for Economic Cooperation and Development [OECD], 2002). Additionally, the extraction process for detection of fentanyl varies based on the sample medium, so direct comparison of fentanyl levels between surface water, soils, sludges, and downstream organisms varies widely due to sample origins and detection methods. The sensitivity and specificity of detection methods for the analyte is also a factor to consider when comparing samples, as different mediums can have different effects on the signal‐to‐noise ratios of sensors (Green et al., 2020; Hontzas, 2024). While test strips and immunoassays exist for fentanyl and some of its analogs, the low levels present in environmental samples would typically require high‐sensitivity detection methods such as tandem mass spectrometry that require significant sample processing and laboratory access, which restricts large‐scale, long‐term sampling projects (Armenian et al., 2018; Ferreira, 2024; Hontzas, 2024). Improving sample processing throughput is likely a key hurdle in overcoming this gap, followed closely by accessibility of sensitive detection equipment.

### 1.3 Sources of fentanyl in the environment

A significant portion of discarded and metabolized drugs ends up in the sewage system before becoming dispersed in the environmental network. Sewage treatment plants are not typically equipped to monitor or remove ECs, so they are subsequently transported to surface waters (Skees et al., 2018), rivers and streams (Mankes & Silver, 2013), and even drinking water (Rodayan et al., 2016). If present in the environment in sufficient quantities, fentanyl's high toxicity may result in adverse effects to aquatic/marine ecosystems, wildlife, and human health. Here we discuss the various introduction points of fentanyl before its dispersal in the environment.

#### 1.3.1 Households

The presence of opioids in surface and wastewater is mainly attributed to human waste following medical or illicit use (Skaggs & Logue, 2022). Households likely contribute a small portion of fentanyl to landfills, primarily through the disposal of used fentanyl products such as patches in the trash, and users of both licit and illicit fentanyl release it into sewer systems via excretion and flushing or disposal down drains, which is considered the primary entry route into the environment (Le Corre et al., 2012; Ternes, 1998). The United States Food & Drug Administration (2020) currently recommends flushing any fentanyl medications that cannot be returned through a take‐back program. According to the College of Pharmacists of British Columbia, a fentanyl patch can still contain >50% of the labeled amount after 3 days of use, and patches are often discarded into the trash or flushed.

#### 1.3.2 Healthcare facilities

Fentanyl is frequently administered in healthcare settings. Healthcare facilities are point sources for pharmaceuticals entering public wastewater (Le Corre et al., 2012), and are often highlighted as major contributors to residues found within the influents of municipal sewage treatment plants (STPs) (Hawkshead, 2008; Le Corre et al., 2012). As with households, this occurs through human excretion and disposal. Although STP pharmaceutical loads vary from hospital to hospital, a study of two New York hospitals in 2008 and 2009 found that fentanyl was the third most common controlled substance that was disposed of through flushing or dumping down drains (Mankes & Silver, 2013).

Studies of hospital effluents have focused primarily on the presence of antibiotics and endocrine disruptors due to their role in the development of antibiotic resistance (Baquero et al., 2008; Williams‐Nguyen et al., 2016) and effects on wildlife and human health (Arcand‐Hoy & Benson, 2009; Encarnacao et al., 2019). Opioids and fentanyl have been monitored in wastewater from a variety of locations, but very few studies have specifically investigated healthcare facility wastewater effluents as sources of fentanyl in the environment (Lin et al., 2010; Oliveira et al., 2015).

#### 1.3.3 Pharmaceutical industries and illicit laboratories

Drug manufacturing facilities produce a large amount of waste that can lead to extremely high levels of pharmaceuticals in their effluents (Fick et al., 2009; Larsson et al., 2007). For example, screening of the effluent from the Patancheru Enviro Tech Ltd. wastewater treatment plant near Hyderabad, India—which receives effluent from approximately 90 bulk drug manufacturers—detected many drugs at levels >100 µg·L^−1^, with some at concentrations higher than their maximal therapeutic human plasma levels (Larsson et al., 2007). The effluent from this plant is eventually discharged into nearby rivers, and the remaining solid waste is transported to a landfill (Fick et al., 2009). In another study, the treated effluent from a Chinese antibiotic factory was found to contain approximately 20 times the human therapeutic plasma level of the antibiotic oxytetracycline (Li et al., 2008). To our knowledge, no studies have investigated the environmental fentanyl contribution from pharmaceutical plants or drug manufacturers; however, based on limited findings from studies such as those mentioned above, it may be presumed that pharmaceutical industries and drug manufacturers that produce fentanyl are contributing relevant waste to the environment.

Similarly, the environmental fentanyl contribution from clandestine laboratories that manufacture fentanyl remains uncharacterized and may be a larger concern considering the increased illicit use in recent years. The illicit manufacture of fentanyl occurs in multiple geographic locations. Currently, many fentanyl precursors are made in China and then transported to Mexico, Caribbean countries, India, the United States, and Canada for transformation into the final product (Pergolizzi et al., 2021). Clandestine laboratories often dispose of chemicals at on‐ and off‐site locations, including household drains, soil, sewage systems, large containers, backyards, roads, waste management facilities, and creeks, resulting in public health risks and environmental contamination (Al‐Obaidi & Fletcher, 2014; Janusz et al., 2003). As fentanyl begins to replace heroin as the illicit opioid of choice in many regions (Reuter et al., 2021), clandestine laboratories and increased distribution of fentanyl may further affect environmental contributions.

A recent document commissioned by the European Monitoring Centre for Drugs and Drug Addiction (now EUDA) recently investigated the environmental impact of synthetic drug production. The authors found residues of drug production waste in surface water and sediments at the site of a remediated drug production waste dump (ter Laak & Emke, 2023). Although fentanyl was not the focus of this study, it emphasizes that the large‐scale production of waste from clandestine operations can have significant impacts on the environment through direction emission into soil and surface waters and through the mixing of these products with standard waste streams. In some instances, drug residues are expected to remain detectable in the groundwater for decades.

A related but little‐studied source of environmental fentanyl stems from people experiencing homelessness. A recent public health report from the Los Angeles County Department of Health in the United States stated that the drug overdose mortality rate among those experiencing homelessness doubled between 2019 and 2021, and that fentanyl has been the main drug type driving these overdose deaths. Of these deaths, those involving fentanyl almost tripled from 20% in 2019 to 58% in 2021 (County of Los Angeles Public Health [LA County Health], 2023), indicating a heavy burden of fentanyl introduced to the environment. Moreover, this population suffers from water and sanitation insecurity and is therefore less likely to make use of the sewer infrastructure (Anthonj et al., 2024), thus creating a source of untraceable environmental introduction that may have impacts on public health and safety.

#### 1.3.4 Agriculture

In addition to direct dumping, ECs can enter soil through irrigation water that comes from effluents or through the use of biosolids as fertilizer (Mohapatra et al., 2016). In most developed countries, effluents are not directly used for crop irrigation, but in many developing countries, this is not the case (Mohapatra et al., 2016). In regions of North America, Europe, Australia, and New Zealand, among others (United Nations: Human Settlements Programme, 2009), biosolids are sometimes used as fertilizers. Biosolids are nutrient‐rich organic materials that are produced from the treatment of domestic sewage wastewater in wastewater treatment facilities and are applied as fertilizer to maintain farmland soil quality. Biosolids are also used for specific applications such as improving soil nutrient content for parks and forests, increasing growth of hybrid poplars, and enhancing the aesthetic value of farmed Christmas trees (Shammas & Wang, 2008). In some instances, if decontamination and testing protocols are not present, this process for biosolid distribution results in the direct application of contaminants to crops, raising reasonable concerns about the effects on the food web (Clarke & Smith, 2011; Mohapatra et al., 2016).

### 1.4 The potential for fentanyl distribution in the environment

As previously discussed, fentanyl and other opioids are being increasingly detected in the environment (Boleda et al., 2007; Skaggs & Logue, 2022). The presence of fentanyl in wastewater and drinking water plants, biosolids, and soils raises concerns. Here, we present an overview of the locations in which fentanyl has been detected, or in which it is likely to be found, based on transport of similar ECs, after its release into the environment.

#### 1.4.1 Wastewater

Wastewater is typically the first stop for fentanyl before dissemination throughout the environment. Although its fate in wastewater is not yet fully understood, its presence is not speculative, as both fentanyl and its primary metabolite, norfentanyl, have been detected in wastewaters around the world (Skees et al., 2018). In fact, wastewater‐based epidemiology (WBE) has been used to monitor fentanyl use in specific populations such as college campuses (Gushgari et al., 2018) and cities with high illicit drug use (Croft et al., 2020; Gushgari et al., 2019). In one prominent example, the city of Cary in North Carolina, in the United States, participated in a pilot program that utilized data collected from a 6‐month WBE study of opioids in sewage water to achieve neighborhood‐level drug use information. This information was then used by the city to inform targeted community outreach programs, which, once implemented, effectively reduced the city's opioid overdoses by 40% (Biobot Analytics, 2022).

Other examples include a 2011 UK study that detected fentanyl in three out of seven wastewater treatment plants (Baker & Kasprzyk‐Hordern, 2011). A follow‐up study in 2013 again detected fentanyl in some of the samples (Baker & Kasprzyk‐Hordern, 2013). Another study analyzed wastewater from 30 cities across China between 2016 and 2017 and found that fentanyl was detected at frequencies of 5% and 4%, respectively (Du et al., 2021). In this case, the low detection frequency was attributed to the low use of fentanyl in the regions sampled, which agreed with surveys estimating the overall consumption of fentanyl is lower in China than in the Americas and Europe (Du et al., 2021). Fentanyl was also detected at trace levels or higher in the wastewater of 12 out of 15 Mexican cities (Cruz‐Cruz et al., 2021). Interestingly, in at least one city, fentanyl was detected more frequently than norfentanyl, which suggested that drug manufacturing, rather than human excretion, was a significant factor (Cruz‐Cruz et al., 2021). In another example, fentanyl, remifentanyl, and alfentanyl were each detected at µg L^−1^ concentrations in both the influent and effluent of wastewater samples from Gauteng, South Africa (Kamika et al., 2021; Kasonga et al., 2021). Fentanyl concentrations in wastewater are typically at low trace levels. Of the studies cited here, concentrations ranged from <0.4 ng·L^−1^ to 25.8 µg L^−1^, none of which can cause acute harm. However, the consequences of chronic exposure to opioids remain unknown (R. Pal et al., 2013; Yadav et al., 2017).

#### 1.4.2 Drinking water and surface water

Fentanyl has also been detected in non‐wastewater environments. A study in Kentucky in the United States in 2018 detected fentanyl in the influent and effluent of a local wastewater treatment plant, as well as the plant's receiving waters, which are used for recreation and also as a source of drinking water for the surrounding community (Skees et al., 2018). A 2015 study of water from a drinking water plant in Ontario, Canada, found fentanyl in all samples (Rodayan et al., 2016). The influent of a drinking water treatment plant in Catalonia, Spain, contained up to 8.5 ng·L^−1^ fentanyl, but potabilization at the plant was able to remove the drug with high efficiency (Boleda et al., 2009). Fentanyl was also found in the tap water of one unnamed Spanish city in 2011 (Rosa Boleda et al., 2011). Fortunately, the low concentrations (≈1.6–8.5 ng·L^−1^ in the studies cited) and infrequent detection of fentanyl in drinking waters indicate a low risk to public health. However, it has been noted that although fentanyl is not frequently detected in drinking waters, it warrants prioritization for future monitoring (Khan & Nicell, 2015), especially because its use is still increasing (Jeffery et al., 2023). A key example of downstream water contamination was demonstrated in 2017, when the Mussel Monitoring Program in Washington State (United States) detected oxycodone in test mussel samples at 1.5 ng·g^−1^, which was a first indicator of opioid contamination in Puget Sound. Unfortunately, it is unlikely that this is an isolated incident, and it is nearly impossible to predict the effect that exposure to multiple co‐contaminants may have on organisms (Taylor, 2019).

#### 1.4.3 Soils, sludges, sediments, and biosolids

Sorption of ECs onto solids and sediments functions as a type of natural attenuation (A. Pal et al., 2010), potentially removing them from circulating waters. However, the hydrophobic properties of fentanyl and the potential for polar‐ionic interactions with clays and soils may result in its concentration within solid wastes and sludges (Kumar et al., 2022; Skaggs & Logue, 2022). In the absence of degradation, this buildup in solids can lead to other potential routes of distribution. For example, the use of contaminated biosolids as fertilizer may affect crops and grazing livestock or wild animals (Pozzebon & Seifert, 2023). Reports on the opioid contents of biosolids are sparse, but recent evidence shows that fentanyl is not completely removed from biosolids (Simpson et al., 2024a). In one study, fentanyl was detected in three different biosolids samples at concentrations ranging from 2.0 to 2.6 µg/kg (Simpson et al., 2024b). Results suggest similar behavior in soils, although only a small number of studies have been performed thus far.

The impacts of net charge, propensity for hydrogen bonding, partition coefficients, dissociation constants, and vapor pressures (Fatta‐Kassinos et al., 2011), as well as the potential for oxidation, biodegradation, or complexation with organics and metals, complicate attempts to determine the sorption of ECs in soil, sludges, and sediments. Although information is sparse, four recent investigations may illuminate some of these otherwise unknown interactions between fentanyl and a natural, solid matrix. Two of these studies investigated the stability and degradation of fentanyl and carfentanil oxalate in four different soil types over the course of 12 weeks (Xega et al., 2020, 2019). After extracting the analytes from the soil samples, neither fentanyl nor carfentanil was detected in the aqueous supernatants, indicating that the analytes remained in the solid phase. Although their concentrations were observed to decrease somewhat over the course of the study, the results suggest that both are stable and immobile in soils.

The third and fourth studies expanded upon the techniques employed by the previous two and spiked fentanyl and fentanyl analogs at 10 µg·g^−1^ and 1 µg·g^−1^ into high clay content soils (Valdez, Rosales, Vu, et al., 2023). Extractions from soil with high clay content are notoriously difficult, but each of the spiked opioids was able to be detected. Research from the same group duplicated the soil spiking experiments in a separate soil and developed a chloroformate extraction process specifically for soil (Valdez, Rosales, Leif, et al., 2023). Unlike the previous investigations, a time course study was not performed, and no real‐world samples were utilized. However, despite limited soil studies thus far, this topic remains an important area of investigation, and the ability to extract and detect fentanyl from complex samples is a critical technological hurdle to overcome in research.

As the application of biosolids and biochar for agricultural fertilizer is common in some countries, it may be prudent to broadly incorporate new methods for decontamination of biosolids, and sample testing to ensure maximal degradation of pharmaceuticals and personal care products before application to crops (Alvarez Ruiz et al., 2024). For example, Australia and New Zealand are known to have a stringent regulatory framework for the production and application of biosolids, which dictates the allowable application rate and the maximum allowable concentration of contaminant chemicals. As such, nearly 70% of their biosolids are used as fertilizer, and the remaining 25%–30% ends up being used on soil for landscaping or landfill purposes, with a minor amount being stockpiled, discharged, or used for other miscellaneous purposes (Australian Water Association, 2020). This growing awareness and vigilant monitoring helps to ensure low levels of fluorinated compounds end up in their food supply and could be adopted by other nations and applied to EC to ensure minimal long‐term risks.

#### 1.4.4 Air and other environments

Fentanyl has also been detected in the air of public transit vehicles and on various surfaces in public areas (Baker et al., 2023). A recent study performed an analysis of 78 air samples collected from public areas such as rooftops, buses, and trains in Washington and Oregon and found that 25% of the public transit locations contained detectable levels of fentanyl. The same study also investigated various surfaces related to public transportation, such as bus seats, vehicle vents, and door handles. Surprisingly, 46% of surfaces sampled had detectable levels of fentanyl. EPA guidelines currently set the acceptable occupational exposure limit for fentanyl in the air to 0.1 µg fentanyl per cubic meter of air, but there are no recommended limits for exposure to fentanyl on surfaces (Baker et al., 2023; United States Environmental Protection Agency, 2018). The authors noted that the levels detected on both surfaces and in the air are not high enough to cause acute harm. However, because long‐term exposure limits are not determined and the effects are unknown, caution and careful adherence to safety precautions are advised for workers who may be chronically exposed.

### 1.5 Additional considerations: Fentanyl precursors, analogs, and degradation and transformation products

Thus far, we have primarily focused on fentanyl. However, fentanyl precursors, metabolites, degradation products, and analogs are all likely to enter the environment in a similar manner and may have their own potential risks and health concerns. Here, we present a brief overview of some of these substances.

#### 1.5.1 Analogs

To circumvent laws and regulations surrounding fentanyl, clandestine labs have synthesized analog compounds, taking advantage of their temporary unregulated nature and the time it takes to recognize and regulate them as controlled substances (Armenian et al., 2018; United States Attorney's Office Central District of California, 2020). And while significant steps have been taken in the United States to schedule all fentanyl‐related substances, many of these analogs continue to be used. The pharmacology, toxicity, bioaccumulation, and persistence of these analogs are not always known, though some exhibit higher toxicity. Carfentanil, for example, is 30–100 times more potent than fentanyl, and furanyl fentanyl and sufentanil are about 7 and 10 times more potent, respectively. Analogs are also increasingly difficult to monitor in the environment, as detection modalities often only detect fentanyl and one or two analogs due to decreased specificity of the detection method when screening for multiple analytes. This issue is further compounded by the continued emergence of synthetic analogs criminals develop and use to avoid regulations (Armenian et al., 2018; Hontzas et al., 2024), which is continually ahead of detection techniques for such analytes.

#### 1.5.2 Precursors

Some fentanyl precursors are also controlled substances (United States Federal Register, 2020). Fentanyl can be synthesized in a variety of ways, but the most common synthesis routes are those developed by Janssen (1965), Siegfried (2004), and Gupta et al. (2005). The chemical precursors N‐phenyl‐1‐(2‐phenylethyl)‐4‐piperidinamine (4ANPP) and N‐phenethyl‐4‐piperidinone (NPP) are frequently used precursors (Mirsafavi et al., 2020), but 4‐anilinopiperidine (4‐AP) and 1‐(tert‐butoxycarbonyl)‐4‐phenylaminopiperidine (boc‐4‐AP) are also used (United Nations Office on Drugs & Crime, 2022). A toxicity assessment of precursors is beyond the scope of this review—however, because significant quantities are produced and sold around the world, their presence within the environment should be considered.

#### 1.5.3 Metabolites

The proportion of consumed fentanyl that remains unaltered after metabolism by the liver is <10%, with most of the drug being converted to the inactive metabolite norfentanyl (Mercadante, 2015; Wilde et al., 2019). Less than 1% of the drug is metabolized to other inactive compounds, including hydroxyfentanyl, hydroxy norfentanyl, despropionylfentanyl (Kuip et al., 2017; Wu et al., 2017), and a variety of minor metabolites (Bista et al., 2014). Norfentanyl has been detected in wastewater samples and can be used to estimate drug use statistics (Gushgari et al., 2018, 2019). Fortunately, most of these metabolites are not active within the body and are therefore unlikely to pose risks to human health if present in the environment at trace levels. However, it is still important to understand the impact of such substances because their transformation and degradation products could potentially affect human and ecosystem health.

#### 1.5.4 Degradation and transformation products

Although the fate of fentanyl and its related compounds in different environmental matrices is not fully understood, recent studies have investigated its degradation and transformation under a variety of conditions. Trawinski et al. (2021) investigated the transformation of fentanyl under simulated sunlight and identified 26 different products, with one of the products believed to be a potentially strong estrogenic compound, and others believed to be mutagenic and developmentally toxic (Trawinski et al., 2021). In a similar study, Blum et al. (2017) exposed a mixture of 30 pharmaceuticals including fentanyl to a ultra violet (UV) mercury lamp for 28 days, and fentanyl degradation was observed with a half‐life between 33 and 122 h, depending on the water matrix. However, in another study, Garg et al. (2010) exposed fentanyl to UV irradiation and a white fluorescent lamp with no degradation detected after 1 week.

Thermal and oxidative degradation of fentanyl may also occur. Interestingly, thermal degradation has been reported across a range of temperatures. Garg et al. studied fentanyl at 350°C and identified norfentanyl and propionanilide as thermal degradants (Garg et al., 2010). Rabinowitz et al. (2004) exposed fentanyl at a temperature of 300°C and observed 30% degradation within 5 min. Fentanyl degradation has also been shown to occur at much lower temperatures. For example, one report described significant fentanyl degradation at 42°C after 4 days, with only 28% of the original drug remaining (Reitstetter & Losser, 2018).

Peroxide and hypochlorite solutions have been used to oxidatively degrade fentanyl with varying degrees of efficiency, with some causing nearly complete degradation after 1 h (Qi et al., 2011). In another study, a system implementing NaBrO_3_ and NaHSO_3_ was used to achieve near‐complete oxidative degradation of fentanyl (Xu et al., 2015). Results suggest that the principal pathway of fentanyl oxidation in the presence of strong oxidizing reagents occurs via N‐dealkylation at the piperidine ring (Lihong H. Pal et al., 2022; Qi et al., 2011). These findings are encouraging, not only because they provide options for future wastewater treatment, but also because, fortunately, many of the products created in this process are not known to be particularly toxic or carcinogenic. However, these studies have relied upon forced degradation, and not all degradation products have been identified. Under normal conditions, the tertiary amine in opioids is highly stable toward hydrolysis, de‐alkylation, and oxidation, which may be an important contributor to their long‐term environmental persistence (H. Pal et al., 2022). A recent study concluded that fentanyl and three other analytes had relatively high stability in river water, but the study was only limited to 6 days and concluded that sorption is likely a key driver of reduced fentanyl levels (Niroula & Pagsuyoin, 2024). Further research is needed to determine fentanyl's potential for degradation in natural environmental conditions and to assess not only its persistence but also any potential toxicity of its degradants.

### 1.6 Persistence, stability, bioaccumulation, and implications for human health

The understanding of fentanyl's fate within the environment would be incomplete without consideration of its persistence, stability, and potential for bioaccumulation and/or biomagnification. Although the literature is sparse, there are clues that can help address some of these questions. In the previously cited study of hospital disposal or flushing of fentanyl and other prescription drugs, the authors also investigated the potential ecological effects of these substances (Mankes & Silver, 2013). They compiled the persistence, bioaccumulation, and toxicity (PBT) indices for each of the drugs and concluded that fentanyl was the least environmentally friendly because it is moderately toxic and may have the potential for bioaccumulation. However, fentanyl's current PBT index indicates that while its presence within the environment is still regarded as moderately toxic, it does not have much potential for bioaccumulation (Janusinfo Region Stockholm, 2018). It should be noted that other opioids have been found in the tissues of aquatic organisms (Duarte et al., 2023), and that PBT index values may change as more fentanyl‐specific studies are conducted.

As discussed previously, some evidence suggests fentanyl may undergo some natural degradation (Niroula & Pagsuyoin, 2024). When distributed or carried to a natural environment, it is expected to partition into soils and sludges and may be relatively stable in those environments. Pharmaceuticals originating from reclaimed wastewater, biosolids, or sewage sludge‐amended soils can enter crops by irrigation and fertilization through the plants’ roots, which can lead to bioaccumulation in different plant parts (Bigott et al., 2020; Grassi et al., 2013). Further research is required to determine if fentanyl, like other pharmaceuticals, can bioaccumulate in the tissues of crops.

## 2 CONCLUSIONS AND FUTURE CHALLENGES

Fentanyl in wastewaters, rivers, streams, and drinking water around the world originates from a combination of sources such as households, houseless populations, healthcare facilities, illicit laboratories, and pharmaceutical companies. Various factors influence environmental levels, such as the baseline consumption of the drug, the size of the surrounding population, rainfall, degradation, dilution, and dispersion into agriculture through biosolid or fertilizer applications. It is therefore likely that levels are underestimated, and as the opioid epidemic continues, fentanyl's presence is likely to increase, making environmental monitoring an emerging critical need.

Current evidence suggests that concentrations of fentanyl in wastewater—and thereby surface and drinking waters—remain below acute toxicity levels. However, this assumption is based on the potency of fentanyl and not necessarily the potency of transformation products. Knowledge of current environmental fentanyl levels is dependent on existing methods to both extract the analyte from highly complex or diluted environmental samples, and then reliably detect those low concentrations. As several examples indicate, determining accurate levels of fentanyl is a challenge because original amounts discharged into the environment are unknown, and quantifying is further compounded by low extraction yields. Additionally, detection methods for fentanyl are still an ongoing area of research as precursors, metabolites, and transformation products continue to emerge, therefore presenting a challenge to either sensitively detect a targeted analyte or detect non‐target transformation products using methods with lower sensitivity.

While our current knowledge base indicates that fentanyl concentrations within the environment remain low, lack of information about bioaccumulation coupled with the high potency of fentanyl and its analogs pose concerns that require further study. Not only are some fentanyl analogs more potent than fentanyl, but it also remains to be seen if any of the environmental transformation products exhibit similar or greater toxicity. As with most other anthropogenically introduced substances, major concerns revolve around the potential effects of chronic exposure to these substances within the environment. Other concerns highlight the potential effects of fentanyl on highly sensitive aquatic organisms and on crops grown in biosolid‐amended soils, which may have human health‐related consequences. The scarcity of sufficient data on the prevalence of fentanyl in the environment and the consequences of long‐term, chronic exposure make it difficult to fully understand the impacts of its growing presence in the environment, emphasizing the need for continued environmental monitoring and research.

## AUTHOR CONTRIBUTIONS

**Deseree J. Tennyson**: Writing—original draft; writing—review and editing. **Kaizad F. Patel**: Writing—original draft; writing—review and editing. **Angela M. Melville**. Writing—review and editing. **Vanessa L. Bailey**: Writing—review and editing. **Kristin M. Omberg**: Conceptualization; funding acquisition; methodology; project administration; resources; supervision; writing—original draft; writing—review and editing. **Loreen R. Lamoureux**: Conceptualization; data curation; methodology; project administration; supervision; writing—original draft; writing—review and editing.

## CONFLICT OF INTEREST STATEMENT

All authors declare no conflicts of interest.
